# Social Reward Responsiveness Moderates the Association between Perceived Social Competence and Depressive Symptoms in Adolescents

**DOI:** 10.1007/s10802-025-01402-1

**Published:** 2026-01-14

**Authors:** Krupali R. Patel, Corinne N. Carlton, Lisa Venanzi, Samantha Pegg, Autumn Kujawa

**Affiliations:** Department of Psychology and Human Development, Vanderbilt University, Peabody College, 230 Appleton Place, Nashville, TN 37203-5721 USA

**Keywords:** Reward responsiveness; depression, Social competence, Adolescents, Electroencephalography

## Abstract

Adolescence is an important social development period that is also marked by increased rates of depression. Positive social interactions are crucial for cultivating social skills and functioning, also known as social competence, and lower social competence has been related to greater depressive symptoms. Still, little is known about factors that impact why some adolescents with low self-perceptions of social competence develop depression while others do not. The present study addressed this gap by examining neural processing of social rewards as a potential moderator of the association between self-perceptions of social competence and depressive symptoms in adolescents. Data from 165 adolescents were collected using electroencephalography during a perceived peer interaction task, eliciting the reward positivity (RewP) event-related potential component in response to social acceptance versus rejection feedback. We identified a moderating effect of social RewP (*b* = 0.52, *p* = .039), such that the negative relation between self-perceived social competence and depressive symptoms was strongest for adolescents with a blunted social RewP. These results provide further evidence for the importance of social reward responsiveness in moderating the relation between risk factors and depressive symptoms in adolescents and highlight the utility of multi-method approaches to examining social processes in depression.

**Supplementary Information:**

The online version contains supplementary material available at 10.1007/s10802-025-01402-1.

## Introduction

Adolescence is a critical period for the development of social skills and the formation of peer relationships (Oudekerk et al., 2015; Rubin et al., 2006; Steinberg et al., 2015), with the importance of peer relationships increasing as adolescents spend more time with peers than their family (Harris, 2001). Peer interactions not only shape current social dynamics but also have long-term implications for mental health outcomes (Barnes et al., 2007; Larson, 2001). Evidence consistently suggests that supportive peer relationships are crucial for positive psychological and socio-emotional development during these formative years (Cubillo, 2022; Hall-Lande et al., 2007; Mitic et al., 2021; Preston & Rew, 2022; Romppanen et al., 2021); on the other hand, interpersonal difficulties are associated with increased risk for depressive symptoms (Altmann & Gotlib, 1988; Beeson et al., 2020). Since social connections are central to adolescent development, a better understanding of individual peer relations could provide key information on the development and maintenance of depression in adolescents.

A lack of positive social experiences during adolescence can impede the development of social skills, which is associated with diminished perceived social competence (i.e., the capacity to demonstrate a range of flexibility in responding to interpersonal interactions; Mitic et al., 2021; Preston & Rew, 2022). One potential explanation for this pattern lies in the role of anticipated peer feedback. Cole (1990, 1991) proposed that peer interactions contribute to a feedback loop whereby negative social encounters (e.g., rejection, criticism, or perceived failure) lead youth to view themselves as socially incompetent. As these negative self-perceptions accumulate, they may contribute to low self-esteem and the development of negative cognitive biases, factors integral to most cognitive models of depression (Cole, 1990, 1991; Cole & Maxwell, 2003; Cole & Turner, 1993). Consistent with this framework, depressed adolescents rate themselves as less socially competent than their non-depressed peers (Altmann & Gotlib, 1988; Marton et al., 1993). This association between self-perceived social competence and depression appears particularly pronounced in adolescence when self-perceptions of social competence are already in flux (Cole et al., 2001; Wigfield et al., 1991). Adolescents who report feeling confident in their ability to interact with and maintain positive relationships with peers are more likely to form new relationships in the future, thereby increasing feelings of belongingness and reducing feelings of loneliness (Sakiz et al., 2021). The endorsement of difficulties in peer interactions, on the other hand, reflects existing self-perception of social incompetence, which is strongly related to depressive symptoms in adolescents (Bédard et al., 2014; Lee et al., 2010), with depressive symptoms also predicting subsequent increases in negative self-perceptions (Kistner et al., 2006; Ohannessian & Vannucci, 2020). Notably, adolescent depression is characterized by lower self-perceptions of social competence but not necessarily differences in peer feedback (Marton et al., 1993). For example, inaccurate perceptions of peer acceptance (i.e., self-perceptions of acceptance not matching peer-reported acceptance) predict increases in depressive symptoms (Kistner et al., 2006).

While self-perceptions of social competence are clearly associated with depressive symptoms, only some adolescents with low self-perceived social competence develop clinical depression. The examination of neurophysiological processes offers insight into why some adolescents with low self-perceived social competence develop more severe or persistent depressive symptoms while others do not. One such process is reward responsiveness, which refers to the brain’s propensity to respond to positive reinforcement (e.g., social acceptance or reward). In adolescents, individual differences in reward responsiveness appear to influence the development of depression in part by moderating the effects of other risk factors such as social stress exposure (Pegg et al., 2019) and parental conflict (Hill et al., 2023). Moreover, adolescents seem to show different patterns of social reward responsiveness than adults (Kwak et al., 2020). Relative to children and adults, adolescents are thought to be particularly sensitive to social feedback, showing increased activation in reward regions of the brain in response to social rewards (Crone & Dahl, 2012). In part due to the salience of peer feedback for depression, social, versus monetary, reward functioning appears particularly impaired in depressed individuals (Forbes & Dahl, 2012; Kujawa, 2024; Zhang et al., 2022).

An event-related potential called the reward positivity (RewP) component derived from electroencephalography (EEG) offers unique insights into reward responsiveness at the neural level (Clayson et al., 2021; Peters et al., 2024). This event-related potential component appears as a relative positive deflection in neural activity in response to positive or rewarding feedback in comparison to negative feedback (Clayson et al., 2021; Peters et al., 2024). RewP peaks around 300 ms after feedback as an enhanced positivity to positive feedback compared to negative feedback. The RewP component was used as the primary index of neural reward responsiveness in the current study based on its established sensitivity to positive social outcomes versus rejection and its functional specificity (Kujawa et al., 2017).

Although RewP has generally been applied in studies of monetary reward processing, RewP can also be applied to capture social reward responsiveness (Kujawa, 2024). Blunted social RewP has been observed as a moderator between depressive symptoms and several key risk factors for internalizing symptoms in adolescents, supporting the idea that altered neural processing of social reward may contribute to underlying vulnerabilities to depressive symptoms in this population (Hill et al., 2023; Nelson et al., 2016; Pegg et al., 2019; D. Zhang et al., 2022). Extending this work, Zhang et al. (2024) identified a subgroup of individuals with melancholic depression who showed a particularly blunted neural response to social reward, compared to healthy and non-melancholic depressed participants, reinforcing the idea that depression is neurobiologically heterogeneous. These findings reinforce the importance of investigating other clinical or cognitive features that may interact with individual differences in reward processing to influence depression risk. Social reward responsiveness may play a key role in the relation between self-perceptions of social competence and depression, as stronger neural responses to positive social interactions may reinforce a sense of competence and self-worth (Morgan et al., 2013). Social RewP may moderate the relation between existing self-perceptions of social competence and depressive symptoms in adolescents such that these associations are stronger for those who tend to show blunted social RewP.

Taken together, while the relation between perceived social competence and depressive symptoms has theoretical and empirical support, explanations for variation between individuals remain unexplored. Adolescents with relatively low social reward responsiveness may be less sensitive to positive social feedback, which could reinforce negative self-perceptions of social competence and strengthen associations between self-perceptions and depressive symptoms. The present study investigated whether social RewP moderates the relation between perceived social competence and depressive symptoms in adolescents. We used a computerized perceived peer interaction task to measure social RewP in adolescents oversampled for depression. Previous work on this task has found it to be moderately believable (Pegg et al., 2019), reliably elicits the RewP, and that individual differences in social RewP magnitude moderate associations between social interactions and affect in real-world contexts (Politte-Corn et al., 2024). Given the role of social reward processing in reinforcing existing self-perceptions of social competence, we hypothesized that the relation between perceived social competence and depressive symptoms in adolescents would be moderated by social RewP, such that adolescents with relatively blunted social RewP would show a stronger negative relation between self-perceived social competence and depressive symptoms, whereas a more enhanced social RewP may weaken the association between self-perceived social competence and depressive symptoms.

## Method

### Participants

A total of 165 eligible participants (61.81% female) aged 14–17 years (*M*_age_ = 15.23, *SD*_age_ = 1.07) at consent, oversampled for current depressive disorders and high risk of depression based on maternal history of depression as determined using a clinical interview at intake, were recruited through advertisements distributed across an academic medical center and the broader community. Flyers and online/email advertisements directed at mothers with teenagers struggling with depression were used to attain a larger sample of depressed adolescents using language such as “Does your child struggle with depression?” or “Are you the mother of a 14–17-year-old? You and your child may be eligible to participate in a research study on adolescence and social behavior.” These advertisements were distributed through a large email listserv to community members interested in research participation, pediatricians’ offices, and social media sites such as Facebook using advertising features. Participants were also recruited through an adolescent depression treatment study (Dickey et al., 2023). Importantly, this treatment study was completed prior to the onset of the COVID-19 pandemic so 52.45% of the currently depressed group was recruited prior to the pandemic, whereas 35.85% of the non-depressed group was recruited prior to the pandemic. Timing of data collection (i.e., before or after implementation of COVID-19 safety precautions) was significantly associated with depressive symptoms, *t*(130.35) = 1.98, *p* =.05, which was expected given that more currently depressed participants were enrolled prior to the pandemic for this treatment study (Dickey et al., 2023). All data reported in this manuscript were collected prior to treatment, and both studies used the same interview and EEG protocols. Adolescents were excluded if they had previous but not current histories of depressive disorders, a history of mania or psychotic disorders unrelated to mood disorder features, autism spectrum disorder or other developmental disabilities, intellectual disabilities, visual or hearing impairments that would interfere with the ability to complete computerized tasks, and/or could not speak English following the exclusion criteria established by the parent study.

Of the 165 enrolled participants, 58 met criteria for major depressive disorder, persistent depressive disorder, or unspecified depressive disorder as determined by the Kaufman Schedule for Affective Disorders and Schizophrenia for School-Age Children–Present and Lifetime (KSADS-PL; Kaufman & Schweder, 2004; K = 1.00) and were considered currently depressed. The remaining 107 participants did not have a history of depressive disorders, with 54 considered high risk due to maternal history of depressive disorders assessed using the Structured Clinical Interview for DSM-5 (First et al., 2016) and 53 considered low risk due to having neither a personal nor maternal history of depressive disorders. Regarding ethnicity and race, 69.7% identified as White, 6.1% as Asian, 16.8% as Black and/or African American, 1.2% as American Indian/Alaskan Native, 1.2% as Native Hawaiian/Pacific Islander, 5.5% as another racial identity, and 6.1% as of Hispanic ethnicity.

Sixty-nine participants were enrolled before COVID-19 social distancing restrictions (from February 2019 to April 2020), and 96 were enrolled after the onset of the COVID-19 pandemic (from October of 2020 through August 2022). Of the 165 enrolled, EEG data were collected for 154 participants. EEG data were excluded from 11 participants due to data quality concerns and three due to outlier scores for social RewP residuals. One participant asked that their data be excluded from analyses. Participants missing EEG data were still included in analyses by employing full information maximum likelihood estimation (Enders & Bandalos, 2001). EEG data were analyzed from 139 adolescents (*M*_age_ = 15.19, *SD*_age_ = 1.09; aged 14–17 [61.23%] female). The final analyzed sample size was 164, as one participant was excluded from analyses since they were missing all primary measures.

### Procedures

All study procedures were reviewed and approved by the Vanderbilt University Institutional Review Board prior to data collectpleted a battery of questionnaires assessing mood and social competence through the REDCap platform and were invited to complete an EEG. The clinical interviews and questionnaire assessments were initially conducted in-person but were transitioned to a teleconference format using Zoom following the onset of the COVID-19 pandemic. The EEG sessions were conducted in person, with appropriate safety precautions implemented during the pandemic.ion. Trained graduate and staff research assistants, supervised by a licensed clinical psychologist (AK), obtained informed consent/assent from both parent and child, and conducted clinical interviews to assess study eligibility. Eligible participants then com

### Measures

#### Depressive Symptoms

Depressive symptoms were assessed using the Mood and Feelings Questionnaire, a 33-item self-report screening tool for 6- to 19-year olds (MFQ; Angold et al., 1995). Participants were asked to rate the extent to which they experienced each item (e.g., “I felt miserable or unhappy” and “I didn’t enjoy anything at all”) over the past two weeks on a scale of 0 (*Not true*), 1 (*Sometimes true*), and 2 (*True*). MFQ items were summed, with higher total scores indicating more severe depressive symptoms. The MFQ has strong internal reliability (Angold et al., 1995; Messer et al., 1995), and for this sample, internal consistency was excellent (α = 0.96). Depressive symptom scores in this sample were right-skewed (skew = 1.19, kurtosis = 0.73), which is similar to other studies using the MFQ (Eg et al., 2018; Sund et al., 2001).

#### Self-Perceived Social Competence

Ratings of perceived social competence were self-reported by adolescents using the social competence subscale of the Self-Perception Profile for Adolescents (SPPA; Harter, 1988). The SPPA is designed to assess various dimensions of self-perception, with the social competence subscale specifically focusing on social skills and peer interactions for youth in grades 8 through 12. This subscale includes five items in the form of paired statements (e.g., “Some teenagers find it hard to make friends BUT Other teenagers find it pretty easy to make friends”). Participants first selected the statement that best described themselves and then indicated whether the selected statement was “*really true”* or “*sort of true”* for them. Scores for each statement range from 1 to 4, where 4 represents the most positive adequate self-judgment and 1 represents the least positive self-judgment. Total self-perceived social competence scores were calculated by taking the average score of the five subscale questions. In this sample, the internal reliability of the social competence subscale was good (α = 0.87).

#### Computerized Peer Interaction Task

During the EEG visit, participants completed a computerized peer interaction task (Kujawa et al., 2014) designed to simulate social interactions between peers. Participants were informed that they would be playing a game with 11 other adolescents also participating in research studies. However, in reality all co-players were fictional with fixed responses. Prior to starting the task, participants provided a photograph for their profile picture. The participant was told their goal was to be one of the six remaining players selected by the group to arrive at the “Big Island of Hawaii” following six rounds of voting on fellow players. Participants also answered some general questions to complete their profile. During each round, participants viewed profiles of other “players” and decided whether to either accept (i.e., “*Keep*”) or reject (i.e., “*Kick Out*”) each co-player. The participant was led to believe that the same co-player was simultaneously voting on them with the same options. To make the task appear more realistic, participants were always presented with the message “waiting for [co-player] to vote” after submitting their vote. After voting time allotted based on pilot data (Kujawa et al., 2014), a fixation cross was shown for 1000 ms, followed by feedback of either an image of a green thumbs up (“*Keep*”) or a red thumbs down (“*Kick Out*”) for 2000 ms. This process was repeated until the participant voted on all co-players left for that round. After each round, a picture of the co-player who did not advance to the next round was shown. In between each round, participants were prompted to answer a new poll question to add information to their profile. They completed this process for a total of six rounds. Importantly, the task was designed so that all participants received roughly equal acceptance and rejection feedback and would always complete the game without being voted out. Due to the deception involved in this task, participants were debriefed at the end of the EEG session and were given the opportunity to request that their data not be used in the analysis. Only one participant asked that their data from this task not be used in the analysis.

Task engagement ratings averaged 3.60, with a standard deviation of 0.86, ranging from 1.33 to 5, on a scale of 1 to 5, including questions such as “I really wanted to stay in the game”, “After a while I lost interest in staying in the game”, and “I would’ve liked to play this game again”. This indicates that participants remained relatively engaged with the task on average and were motivated by the interactions with perceived peers. An example trial for this task can be found in the Supplementary Materials (Figure S1).

### EEG Data Collection and Processing

EEG data were continuously recorded using a 32-channel BrainProducts antiCHamp system (Munich, Germany BrainVision Recorder, 2020), based on the standard 10/20 layout. Electrooculogram (EOG) measurements from eye movements were collected with facial electrodes placed approximately 1 cm above and below the right eye and 1 cm to the side of each outer eye corner. Per the BrainProducts bipolar-to-auxiliary adapter design, bipolar electrodes were referenced to a ground electrode on the back of the participant’s neck. Impedances were reduced to below 30 kΩ, and active electrode voltages were referenced online to Cz. A 24-bit resolution and sampling rate of 1000 Hz were used to digitize the recordings.

Data were processed offline using BrainVision Analyzer (BrainProducts, Munich, Germany). Data were first passed through a band-pass filter with cutoffs of 0.1 Hz and 30 Hz and then re-referenced offline to mastoid electrodes TP9 and TP10. Trials were segmented from continuous EEG data from − 200 ms before to 1000 ms after feedback. Ocular correction was applied using Gratton’s algorithm (Gratton et al., 1983). Data from channels that had low trial counts (< 25% of total trials possible available at a given channel) were interpolated prior to artifact rejection. Following segmentation and ocular correction, semiautomatic artifact rejection was employed with the following criteria: a maximal voltage step of 50 µV between sample points, within trial maximum voltage difference of 175 µV in intervals of 400 ms, minimal amplitude of −200 µV and maximal allowed amplitude of 200 µV, and 0.5 µV lowest allowed activity in intervals of 100 ms. Visual inspection was conducted to remove any remaining artifacts. Segments were averaged by feedback type (acceptance or rejection), and baseline corrected from − 200 ms to 0 ms before feedback onset.

Vertical EOG (VEO) and horizontal EOG (HEO) electrodes placed on the face were used for ocular correction. To minimize close contact with participants who completed EEGs relatively early in the COVID-19 pandemic, before vaccines were available, only 16 channels were used for data collection. We analyzed the electrodes included in both montages (Cz) and the number of channels used for data collection was not related to RewP magnitude, *t*(27.05) = −0.57, *p* =.57. Additionally, due to COVID-19 safety protocols and/or difficulty obtaining a usable signal, some participants’ facial EOG electrodes were replaced with cap electrodes for ocular correction. When facial electrodes were either not placed or were unusable, cap electrode FP1 with common reference was used to measure vertical eye movement and FT9 with FT10 as reference was used for horizontal eye movement measurements. For ocular correction, both sets of facial electrodes were used for 78 participants; 34 had either VEO or HEO replaced with scalp electrodes for ocular correction procedures (described below), and 42 had both VEO and HEO replaced with scalp electrodes. Previous results have shown that replacing facial electrodes with cap electrodes has minimal effects on RewP scores (Pegg et al., 2024). In this sample, RewP magnitude did not differ based on which electrodes were used for ocular correction, *t*(136.69) = −0.28, *p* = .78. Nonetheless, a binary indicator of ocular correction electrodes (i.e., using both VEO and HEO versus replacing 1 or both with a scalp electrode) was included in all primary models. We also tested models covarying the use of both, one, or no facial electrodes for ocular correction and present results within each ocular correction electrodes subgroup in the Supplementary Materials (Tables S3 and S4).

The RewP was scored as the mean amplitude at Cz in the 275–375 ms window following acceptance feedback compared to rejection, which is consistent with prior work on social RewP with the computerized peer interaction task (Hill et al., 2023; Long et al., 2024; Politte-Corn et al., 2024; Rappaport et al., 2019). Residual scores were calculated to isolate responses to acceptance relative to rejection feedback. First, a paired samples *t*-test was used to test whether social RewP amplitude varied as a function of peer feedback (i.e., if social RewP differed significantly between the acceptance and rejection conditions). As expected, social RewP was enhanced to acceptance versus rejection, *t*(141) = 6.77, *p* <.001. Next, unstandardized residuals from a linear regression model predicting social RewP to acceptance, accounting for the social RewP to rejection, were calculated and saved for analysis. A residual-based approach is preferred to a subtraction approach since the residual better isolates condition-related neural activity by controlling for overlapping signals that are present for both, allowing for the detection of components specific to the condition of interest (e.g., reward processing; Meyer et al., 2017).

#### Data Analysis

To examine associations between key variables, Spearman bivariate correlations were conducted between sex, depressive symptom severity, perceived self-competence, and unstandardized RewP residuals to social reward. Then, multiple linear regressions were conducted using the *lavaan* package in *R* (Rosseel, 2012) to test the main and interactive effects of sex, ocular correction electrodes, RewP residuals to social reward, and perceived social competence on depressive symptom severity. To account for the non-normal distribution of residuals for depressive symptoms, models were estimated using robust maximum likelihood estimation.

Analyses of missing data showed that teen race was significantly associated with missing EEG data, *X*^*2*^ (5, *N* = 163) = 13.30, *p* =.02, with 34.62% of Black participants and 20.00% of Asian participants missing EEG data for this task. In comparison, 8.70% of White participants had their EEG data excluded. Teen sex, *X*^*2*^ (1, *N* = 164) = 1.49, *p* =.70, teen age, *t*(28.21) = −1.09, *p* =.29, teen ethnicity, *X*^*2*^ (1, *N* = 164) = 1.23, *p* =.27, and depressive symptoms, *t*(27.38) = −1.41, *p* =.17, were not significantly associated with missing EEG data. Missing data were handled using full information maximum likelihood (FIML; Enders & Bandalos, 2001), which obtains unbiased estimates of model parameters by using all available data points. Significant interactions were interpreted with simple slopes analyses using the interactions R package (Long, 2024) to account for the potential moderating effect of sex on the relation between social competence and social RewP. Regions of significance were evaluated using the Johnson-Neyman technique (Johnson & Neyman, 1936).

Sex was included in the model given its potential influence on depressive symptoms, as depression is more prevalent in female adolescents (Dalsgaard et al., 2020; Yzerbyt et al., 2004) as well as previous research showing potential sex-based differences in RewP where boys showed larger RewPs than girls (Bunford et al., 2022). Girls may also show greater vulnerability for depressive symptoms based on peer support levels at an earlier age than boys (Bédard et al., 2014), emphasizing the importance of looking at sex when investigating the relation between social competence and depressive symptoms. Pubertal development and age were also tested as exploratory covariates in our adolescent population due to previous studies indicating that both depression (Gupta et al., 2024; Stumper & Alloy, 2023) and reward processing (Barendse et al., 2024; Ladouceur et al., 2019) are impacted by both processes. Full results from the investigation of age and puberty as covariates can be found in the Supplementary Materials (Table S2).

We also included ocular correction electrodes (i.e., whether facial electrodes were replaced with scalp electrodes for detecting eye movements) as a covariate to account for potential effects of method differences.

## Results

### Bivariate Correlations

Bivariate correlation analyses between study variables are presented in Table 1. Greater depressive symptoms were moderately correlated with lower self-perceived social competence, *r*(161) = − 0.47, *p* <.001. The use of scalp electrodes for ocular correction instead of exclusively facial electrodes was associated with lower depressive symptoms, *r*(151) = − 0.23, *p* <.01, likely due to more of the depressed group being enrolled pre-pandemic when facial electrodes were applied. We also explored correlations with EEG method differences, COVID-19 changes, and pubertal development. Results from these analyses are presented in Table S1.

**Table 1:** Descriptive statistics and bivariate correlations (Pearson’s r) between study variables

Measure	Mean (SD)	1	2	3	4
1. Sex (% female)	62.20%	-			
2. Self-Perceived Social Competence	2.79 (0.81)	−0.10	-		
3. Social RewP (Residual)	0.00 (4.83)	−0.14	0.10	-	
4. Depressive Symptoms	16.34 (14.76)	0.12	−0.47***	−0.14	-

*p <.05, **p <.01, ***p <.001

### Main Effects

Multiple linear regressions were then computed to examine unique predictors of depressive symptoms, controlling for sex and ocular correction electrodes (Table 2). Lower perceived social competence was associated with greater depressive symptoms with and without controlling for sex. There was no main effect of social RewP on depressive symptoms.

**Table 2:** Multiple regression models predicting depressive symptoms

Predictor	*b* (SE)	β	*b* (SE)	β
	Model 1		Model 2	
Intercept	40.47 (5.44)***	2.75	39.89 (5.85)***	2.71
Teen Sex	1.29 (2.12)	0.04	−1.00 (2.10)	0.03
Ocular Correction Electrodes	−6.34 (2.07)**	−0.22	−6.64 (1.98)**	−0.23
Self-Perceived Social Competence	−8.28 (1.30)***	−0.45	−7.94 (1.29)***	−0.43
Social RewP (Residual)	−0.25 (0.23)	−0.08	−1.77 (0.69)**	−0.58
Self-Perceived Social Competence X Social RewP (Residual)	-	-	0.55 (0.27)*	0.52
*R*^*2*^	0.27		0.30	

*p <.05, **p <.01, ***p <.001

### Moderation Analyses

**Fig. 1:**
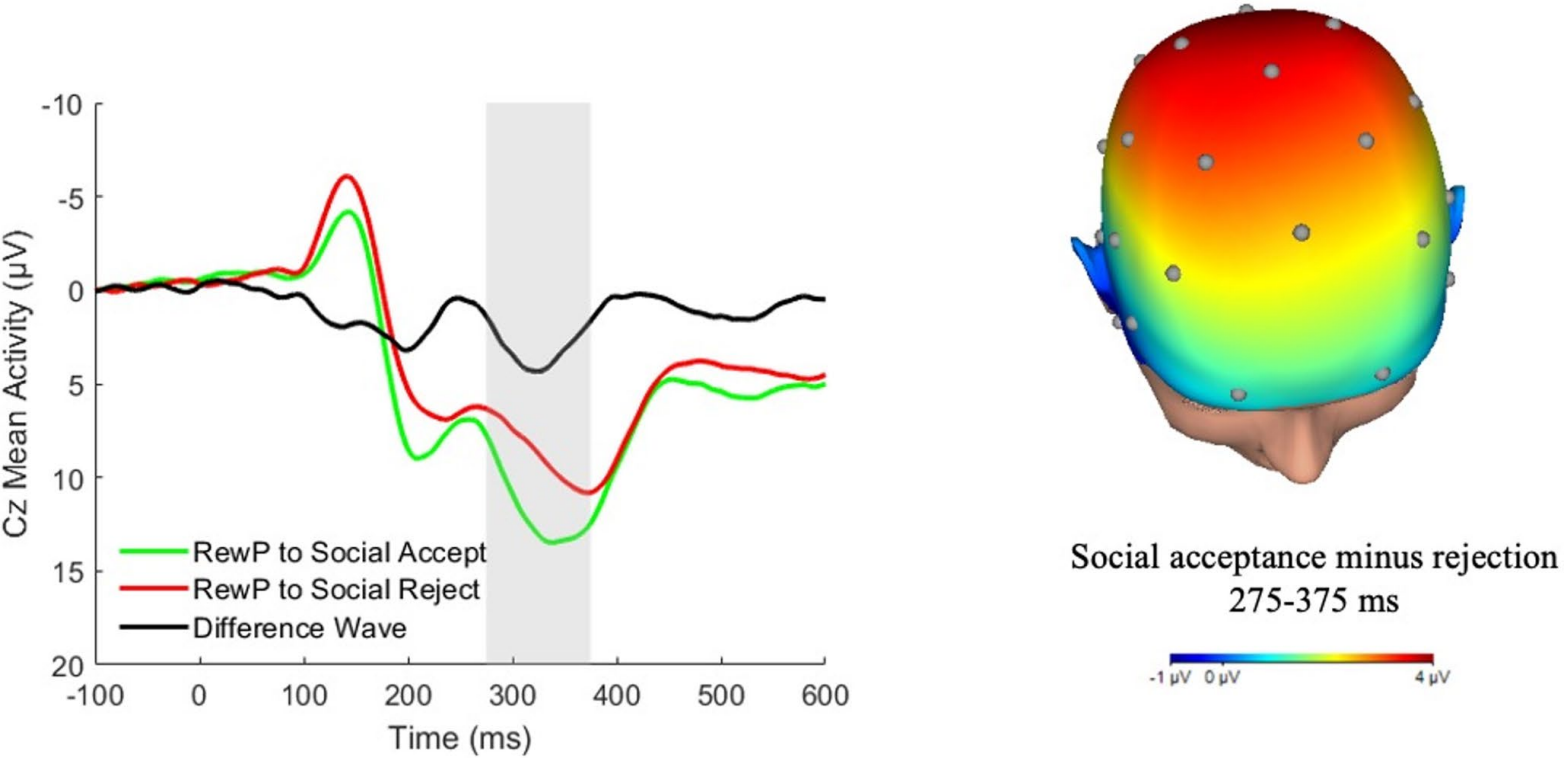
Grand Average ERP Waveforms of response to social acceptance and social rejection feedback at Cz electrode (negative plotted up), and scalp distribution of the difference between RewP to social acceptance and RewP to social rejection 275-375ms after feedback

**Fig. 2:**
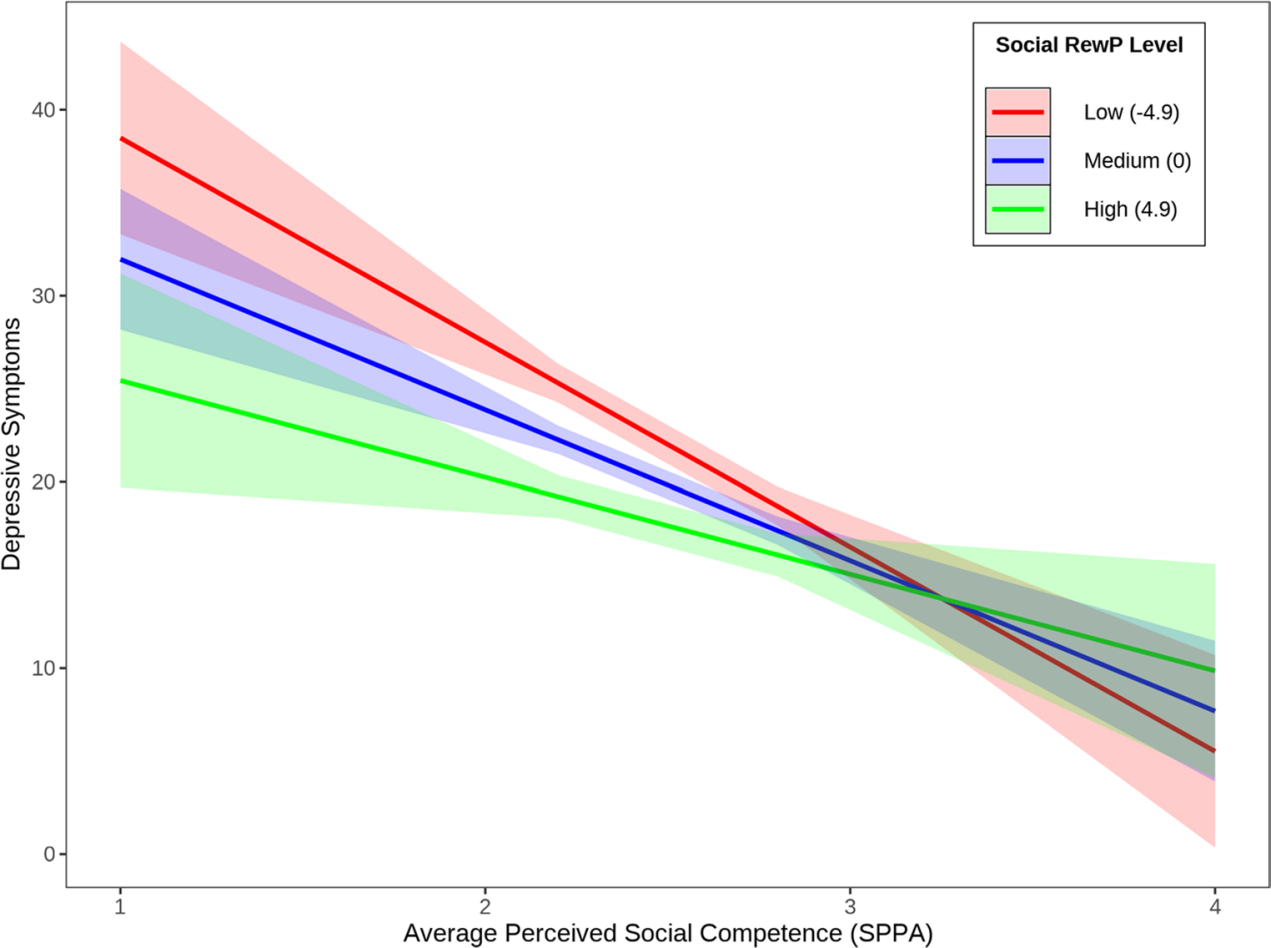
Conditional Effects of Depressive Symptoms for Social RewP by Perceived Social Competence

The interaction of perceived social competence with social RewP was added to step 2 of the regression model (Table 2; Fig. 1). Social RewP significantly moderated the relation between perceived social competence and depressive symptoms when controlling for sex and ocular correction electrodes, *b* = 0.52, *p* =.04, *R*^*2*^ = 0.30. Simple slopes were analyzed at low (1 SD below the mean), mean, and high (1 SD above the mean) values of social RewP to interpret the interaction (Fig. 2). The effect of perceived social competence on depressive symptoms was significantly negative at low (*b* = −10.60, *SE* = 1.71, *p* <.001), mean (*b* = −7.94, *SE* = 1.29, *p* <.001), and high (*b* = −5.27, *SE* = 1.97, *p* <.01) levels of social RewP, with the effect size of self-perceived social competence on depression decreasing as social RewP increases. Further, Johnson-Neyman analyses showed that the relation between perceived social competence and depressive symptoms was significant for social RewP values below 6.21 µV, but at higher levels of social RewP, the effect was no longer significant.

### Supplemental Analyses

Finally, we conducted exploratory analyses to test the impacts of age/puberty, methods differences, the COVID-19 pandemic, and anxiety disorders on results. Age and pubertal development were tested as correlates of key study variables and covariates in regression models (Tables S1 and S2 in Supplementary Materials). Primary results remained significant when accounting for both variables. Although we covaried whether either facial ocular correction electrode was replaced in primary analyses, we also tested models within each specific combination of ocular correction electrodes to test the robustness of results. The interaction between self-perceived social competence and RewP to social reward was only significant when scalp electrodes were used for ocular correction (Table S3), but the general direction of the effects remained consistent across all three types of ocular correction electrodes (Table S4). When considering enrollment timing (e.g., before or during COVID-19), we found that although enrollment timing was significantly related to RewP residuals and depressive symptoms (Table S1), the interaction between self-perceived social competence and RewP to social acceptance remained significant when controlling for whether EEG data were collected before or during COVID-19 (Table S5). Lastly, given the comorbidity between anxiety and depression, we tested models covarying for common anxiety diagnoses (Table S6). The interaction between self-perceived social competence and RewP to social acceptance remained significantly associated with depression when controlling for current social anxiety disorder but not generalized anxiety or panic disorder (β = 0.49, *p* =.040), with the relation between self-perceived social competence and depressive symptoms remaining the most negative at lower levels of social RewP (*b* = −9.93, *p* <.001). See Supplementary Materials for more details on these results and interpretation.

## Discussion

We examined associations of social reward responsiveness (measured by the social RewP), self-perceived social competence, and depressive symptoms in adolescents oversampled for depression. Consistent with prior work (Bédard et al., 2014; Cole, 1991; Cole et al., 2001; Lee et al., 2010), lower self-perceived social competence was associated with depressive symptoms. We did not observe a main effect of social RewP on depressive symptoms, which is in line with previous research that found no direct association between social RewP and depressive symptoms (Hill et al., 2023; Pegg et al., 2019; Politte-Corn et al., 2024). Instead, as hypothesized, social RewP moderated the relation between self-perceived social competence and depressive symptoms in adolescents, such that at lower levels of social RewP, the association between self-perceived social competence and depressive symptoms was relatively stronger. These results extend prior work investigating the potential moderating role of social RewP on the relation between risk factors of depression and the development of depressive symptoms in adolescents.

This study was the first to examine the role of neural reactivity to social reward in the relation between self-perceived social competence and depressive symptoms in adolescents. As with prior work, we observed a robust relation between self-perceived social competence and depressive symptoms (Altmann & Gotlib, 1988; Bédard et al., 2014; Cole et al., 2001; Epkins & Seegan, 2015; Marton et al., 1993; Ohannessian & Vannucci, 2020). While previous research identified additional factors, such as stress exposure (Cohen et al., 2015), that influence this relation, this study is the first to investigate the role of reward-related neural functioning on the relation between self-perceived social competence and depressive symptoms. In this study and others (e.g., Pegg et al., 2019) blunted social RewP appears to exacerbate the relation between risk factors and depressive symptoms. These findings align with recent work by Zhang et al. (2024), who identified that a subgroup of individuals with melancholic depression exhibited an especially blunted neural response to reward, highlighting the role of aberrations in reward processing in different presentations of depression.

These findings also add to a growing body of literature investigating reward-related factors that influence the development of depression by identifying a novel, social neural moderator of the relation between self-perceived social competence and depressive symptoms in adolescents. Adolescents with relatively lower social reward responsiveness had a more substantial negative association between existing self-perceptions of social competence and depression, which could reflect a reinforcement of negative self-perceptions due to blunted sensitivity to positive social feedback. Understanding the role of social reward responsiveness in the development of depressive symptoms in adolescents may open the door for interventions targeting specific emotional processes (Kujawa, 2024). Specifically, the inclusion of neurophysiological measures of reward responsiveness in this study enabled the identification of a potential moderator underlying this relation, highlighting social RewP as a factor that may influence the developmental trajectory of depressive symptoms in adolescents with low self-perceived social competence.

During adolescence, both self-perceptions (Cole et al., 2001; Wigfield et al., 1991) and reward-related brain function (Davey et al., 2008) are in flux, creating conditions ripe for potential dysfunction. Adolescents experiencing low self-perceptions of their social competence and blunted social reward responsiveness may experience continuous reinforcement of their negative self-perceptions without corresponding strong neural responses to positive reward that could instead reinforce a sense of self-efficacy and self-worth. The combination of low self-perceived social competence and low social reward responsiveness may be particularly detrimental, as these adolescents may both perceive fewer social successes and have weaker neural responses to successes, leading to inaccurate perceptions of social failure, limited corrective experiences, and increasing vulnerability to depressive symptoms.

Identifying unique treatment targets for this population could pave the way for the development of more effective early interventions for depression. In adults, brief motivation manipulations have shown promise for increasing blunted RewP (Pegg & Kujawa, 2020), and interventions targeting deficits in reward sensitivity have been more effective at increasing positive affect (Craske et al., 2019) and improving clinical status (Craske et al., 2023) than standard negative emotion-focused interventions. Interventions that aim to enhance sensitivity to positive social feedback or reduce the impact of negative peer experiences may be particularly beneficial for adolescents with low self-perceived social competence and blunted social RewP. Additionally, social skills training programs that directly improve social competence, such as interpersonal psychotherapy for adolescents (Filia et al., 2021) or Interpersonal Effectiveness Skills Training (Atta et al., 2024) could be useful in diminishing depressive symptoms or increasing self-perceived social competence, particularly for adolescents with blunted social RewP. Future research should explore whether these social competence-based interventions can enhance social RewP in adolescents at risk for the development of depressive symptoms.

Additional studies that manipulate risk of social reward or rejection could help clarify the link between social RewP, self-perceived social competence, and depressive symptoms. Specifically, research aimed at understanding turning points in the positive feedback loop between self-perceived social competence and the development of depressive symptoms could contribute to enhancing treatments targeting social competence in adolescents. Social rewards that are identified as especially salient to adolescents (i.e., conditions of rejection or acceptance they are especially sensitive to) could provide important context for reinforced self-perceptions contributing to depressive symptoms.

There are several key limitations of the present study. First, causal inferences cannot be drawn regarding the relation between self-perceived social competence, social RewP, and depressive symptoms due to the study’s cross-sectional nature. Future longitudinal research is needed to determine whether blunted social RewP prospectively predicts depressive symptom trajectories in adolescents with low self-esteem. Second, although theoretical justification exists for the study of adolescent self-perceived social competence, previous evidence indicates that secondary measures of social competence (e.g., parent- or peer-rated) may also influence the development of depressive symptoms in adolescents (Altmann & Gotlib, 1988; Barnes et al., 2007; Beeson et al., 2020). Future research should aim to determine whether the effect of social RewP differs from the current findings in the relation between observed (e.g., parent or peer report) social competence and depressive symptoms. Third, although the computerized peer interaction task did elicit differences in neural responses for acceptance and rejection, it was unable to completely mirror the complexities of social interactions. Additional research should aim to assess and increase the ecological validity of this task. One method for doing so could be using ecological momentary assessment to compare real-world social functioning and resulting affect with in-lab assessments. The present study was additionally limited by less EEG data from racially minoritized participants, which is becoming more commonly reported with increased awareness of disparities in EEG research and limits the generalizability of related findings (Choy et al., 2022). Improvements in EEG technology are needed to facilitate quality data collection that minimizes participant burden for those with a diverse range of hair types and styles.

This study provides insight into the impact of social reward responsiveness on the relation between self-perceptions of social competence and the development of depressive symptoms in adolescents. Consistent with prior research, lower self-perceived social competence was associated with greater depressive symptoms. Although social RewP was not directly related to depressive symptoms, it moderated the relation between self-perceived social competence and depression, suggesting that blunted social RewP may exacerbate vulnerability to depressive symptoms. These findings contribute to growing evidence that low social reward responsiveness plays a key role in adolescent depression and may be a potential target for intervention. Specifically, programs designed to enhance sensitivity to social reward and feedback or improve social competence may mitigate depressive symptoms in adolescents with low self-perceptions of social competence.

## Supplementary Information

Below is the link to the electronic supplementary material.

ESM 1 DOCX (146 KB)
