# Supplementary material for: Social Reward Responsiveness Moderates the Association between Perceived Social Competence and Depressive Symptoms in Adolescents
Source: Res Child Adolesc Psychopathol. 2026 Jan 14;54(1):7. doi: 10.1007/s10802-025-01402-1 (PMC12804202; doi:10.1007/s10802-025-01402-1)
Supplement: Supplementary file 1 — DOCX (146 KB) [file 10802_2025_1402_MOESM1_ESM.docx]

Supplementary Information

Supplementary Results

We also tested age and pubertal development as correlates of key study variables and covariates in regression models (Tables S1 and S2 in Supplementary Materials). Primary results remained significant, accounting for both. Given variability in electrodes used for ocular correction procedures, we explored analyses in each subgroup, partitioned by ocular correction electrodes (Tables S3 and S4). The moderating effect of social RewP remained significant for the subgroup who had at least one facial electrode replaced (N = 78), but not for those where both facial electrodes were used (N = 77). Further probing of the interactions in these models showed that although the interaction was no longer significant when only considering those where both facial electrodes were used for ocular correction (Table S3), the general direction of the effects remained consistent (Table S4).

Although EEG method differences (ocular correction electrodes and electrode number) are confounded with the onset of the COVID-19 pandemic, we also tested models covarying whether participants were enrolled pre- or during the pandemic (Table S5). Though COVID-19 timing was significantly related to EEG data and depression (Table S1), the interaction of interest remained significant when controlling for whether EEG data were collected before or during COVID-19 (Table S5).

Lastly, given the comorbidity between anxiety and depression, we tested models covarying for common anxiety diagnoses (Table S6). Specifically, 23.8% of the sample met criteria for current social anxiety disorder, 16.5% current generalized anxiety disorder, and 1.8% current panic disorder. When presence of current generalized anxiety disorder at intake was included as a covariate in models testing for a potential moderating effect of social RewP on the relation between self-perceived social competence and depressive symptoms, the interaction effect was no longer significant (β = 0.30, *p* = .23). When controlling for current social anxiety disorder, the primary interaction remained significant (β = 0.49, *p* = .040), with the relation between self-perceived social competence and depressive symptoms remaining the most negative at lower levels of social RewP (*b* = -9.93, *p* < .001). The persistence of the interaction when controlling for social anxiety, but not generalized anxiety disorder, may be in part due to an overlap between generalized anxiety and depressive symptoms (Zbozinek et al., 2012).

Table S1

Bivariate Correlations (Pearson's r) Between Study Variables, Puberty, Teen Age, and COVID-19 timing (Spearman)

| Measure | 1 | 2 | 3 | 4 | 5 | 6 | 7 | 8 |
| --- | --- | --- | --- | --- | --- | --- | --- | --- |
| 1. Sex (% female) | - |  |  |  |  |  |  |  |
| 1. Age | -0.04 | - |  |  |  |  |  |  |
| 1. Pubertal Status^a^ | 0.51*** | 0.38*** | - |  |  |  |  |  |
| 1. Number of Electrodes^b^ | 0.06 | -0.03 | 0.10 | - |  |  |  |  |
| 1. Ocular Correction Electrodes^b^ | -0.10 | 0.13 | -0.06 | -0.41*** | - |  |  |  |
| 1. COVID-19 Timing^c^ | -0.18 | 0.06 | -0.09 | -0.27** | 0.60*** | - |  |  |
| 1. Self-Perceived Social Competence | -0.10 | -0.03 | -0.26** | 0.06 | -0.02 | -0.10 | - |  |
| 1. Social RewP (Residual) | -0.14 | -0.11 | -0.11 | 0.05 | 0.02 | 0.18* | 0.10 | - |
| 1. Depressive Symptoms | 0.12 | 0.10 | 0.29*** | 0.19* | -0.23** | -0.16* | -0.47*** | -0.14 |

Note: **p* < .05, ***p* < .01, ****p* < .001. ^a^Pubertal development measured using the self-administered Puberty Development Scale (Carskadon & Acebo, 1993). ^b^Number of electrodes indicates 16 versus 32 electrodes used for EEG data collection. ^c^Ocular Correction Electrodes: 0 = facial electrodes used, 1 = at least 1 facial electrode replaced. ^c^COVID -19 Timing: 0 = before data collection pause in April 2020, 1 = after data collection resumed in October 2020.

Table S2

Multiple Regression Models Predicting Depressive Symptoms, Controlling for Pubertal Development and Age

| Predictor | *b* (SE) | β | *b* (SE) | β |
| --- | --- | --- | --- | --- |
|  | Model 1 | | Model 2 | |
|  |  |  |  |  |
| Intercept | 17.40 (15.27) | 1.18 | 17.46 (15.09) | 1.19 |
| Teen Sex | -1.37 (2.51) | -0.05 | -1.62 (2.48) | -0.05 |
| Ocular Correction Electrodes | -6.58 (2.06)** | -0.22 | -6.86 (2.04)** | -0.23 |
| Pubertal Development^a^ | 4.99 (2.49)* | 0.18 | 4.93 (2.45)* | 0.18 |
| Teen Age | 0.57 (1.06) | 0.04 | 0.54 (1.04) | 004 |
| Self-Perceived Social Competence | -7.58 (1.32)*** | -0.41 | -7.25 (1.32)*** | -0.40 |
| Social RewP (Residual) | -0.23 (0.22) | -0.07 | -1.73 (0.77)* | -0.56 |
| Self-Perceived Social Competence X Social RewP (Residual) | - | - | 0.54 (0.27)* | 0.51 |
| *R^2^* | 0.30 |  | 0.32 |  |

Note. *p < .05, **p < .01, ***p < .001. ^a^Pubertal development measured by the self-rated Pubertal Development Scale (Petersen et al., 1998), where items are rated on a 4-point scale with 1, no development; 2, beginning development; 3, additional development; and 4 development already passed.

Table S3

Multiple Regression Models Predicting Depressive Symptoms, Separated by Ocular Correction Electrodes

| Predictor | *b* (SE) | β | *b* (SE) | | β | *b* (SE) | | β |
| --- | --- | --- | --- | --- | --- | --- | --- | --- |
|  | Facial Electrodes Only (VEO and HEO; *n* = 78) | | One Facial and One Scalp Electrode (VEO or HEO; *n* = 35) | | | Scalp Electrodes Only (*n* = 42) | | |
| Intercept | 46.96 (9.33)*** | 2.80 | 33.36 (9.94)** | | 2.83 | 21.48 (6.73)* | | 2.02 |
| Teen Sex | -0.80 (3.71) | -0.02 | -1.39 (4.04) | | -0.06 | 5.98 (2.85)* | | 0.26 |
| Self-Perceived Social Competence | -9.38 (2.11)*** | -0.47 | -6.46 (2.80)* | | -0.37 | -6.75 (1.66)*** | | -0.51 |
| Social RewP (Residual) | -1.35 (1.48) | -0.37 | -2.44 (1.46) | | -1.13 | -1.99 (0.92)* | | -0.79 |
| Self- Perceived Social Competence X Social RewP (Residual) | 0.35 (0.50) | 0.29 | 0.80 (0.53) | | 1.01 | 0.70 (0.32)* | | 0.80 |
| *R^2^* | 0.25 | | 0.26 |  | | 0.45 |  | |

Note. *p < .05, **p < .01, ***p < .001.

Table S4

Simple Slopes Analysis of Regression Interaction Between Self-Perceived Social Competence and Social RewP, Separated by Ocular Correction Electrodes

| Moderator Level | *b* (SE) | *p* |
| --- | --- | --- |
| Facial Electrodes Only (VEO and HEO) |  |  |
| Low RewP (-4.61) | -11.02 (2.86) | < .001 |
| Average RewP (0) | -9.38 (2.11) | < .001 |
| High RewP (4.61) | -7.75 (3.36) | .024 |
| One Facial and One Scalp Electrode (VEO or HEO) |  |  |
| Low RewP (-5.6) | -10.92 (3.92) | .010 |
| Average RewP (0) | -6.46 (2.80) | .028 |
| High RewP (5.6) | -2.01 (4.22) | .638 |
| Scalp Electrodes Only |  |  |
| Low RewP (-4.59) | -9.98 (2.07) | < .001 |
| Average RewP (0) | -6.75 (1.66) | < .001 |
| High RewP (4.59) | -3.52 (2.35) | .144 |

Table S5

Multiple Regression Models Predicting Depressive Symptoms, controlling for COVID-19 Timing (pre or during)

| Predictor | *b* (SE) | β | *b* (SE) | β |
| --- | --- | --- | --- | --- |
|  | Model 1 | | Model 2 | |
|  |  |  |  |  |
| Intercept | 42.39 (5.73) | 2.88 | 42.17 (5.65)*** | 2.87 |
| Teen Sex | 1.01 (2.12) | 0.03 | 0.71 (2.10) | 0.02 |
| Ocular Correction Electrodes | -2.65 (1.51) | -1.53 | -2.72 (1.49) | -0.16 |
| COVID-19 Timing | -2.90 (2.60) | -0.19 | -3.28 (2.58) | -0.11 |
| Self-Perceived Social Competence | -7.07 (1.08)*** | -0.48 | -8.34 (1.39)*** | -0.45 |
| Social RewP (Residual) | -0.83 (1.12) | -0.06 | -1.95 (0.79)* | -0.65 |
| Self-Perceived Social Competence X Social RewP (Residual) | - | - | 0.63 (0.28)* | 0.60 |
| *R^2^* | 0.28 |  | 0.31 |  |

Note. *p < .05, **p < .01, ***p < .001.

Table S6

Multiple Regression Models Predicting Depressive Symptoms, Controlling for Current Anxiety Disorders

| Predictor | *b* (SE) | β | *b* (SE) | | β | *b* (SE) | | β |
| --- | --- | --- | --- | --- | --- | --- | --- | --- |
|  | Generalized Anxiety Disorder (*n* = 39) | | Social Anxiety Disorder (*n* = 27) | | | Panic Disorder (*n* = 3) | | |
| Intercept | 35.19 (4.93)*** | 2.39 | 36.92 (5.57)*** | | 2.51 | 39.74 (5.40)*** | | 2.70 |
| Teen Sex | -0.57 (1.91) | -0.02 | 1.11 (2.06) | | 0.04 | 0.99 (2.10) | | 0.03 |
| Ocular Correction Electrodes | -6.26 (1.84)** | -0.21 | -6.25 (2.02)** | | -0.21 | -6.65 (2.05)** | | -0.23 |
| Current Anxiety Disorder^a^ | 13.09 (2.27)*** | 0.38 | 4.95 (2.96) | | 0.13 | 3.33 (6.47) | | 0.04 |
| Self-Perceived Social Competence | -6.49 (1.20)*** | -0.37 | -7.29 (1.34)*** | | -0.40 | -7.89 (1.30)*** | | -0.43 |
| Social RewP (Residual) | -1.13 (0.71) | -0.37 | -1.69 (0.77)* | | -0.55 | -1.67 (0.80)* | | -0.55 |
| Self- Perceived Social Competence X Social RewP (Residual) | 0.30 (0.25) | 0.28 | 0.55 (0.27)* | | 0.51 | 0.52 (0.28) | | 0.49 |
| *R^2^* | 0.42 | | 0.32 |  | | 0.30 |  | |

Note. *p < .05, **p < .01, ***p < .001. ^a^Each model includes all 164 subjects.

Figure S1

Example trial in the computerized peer interaction task


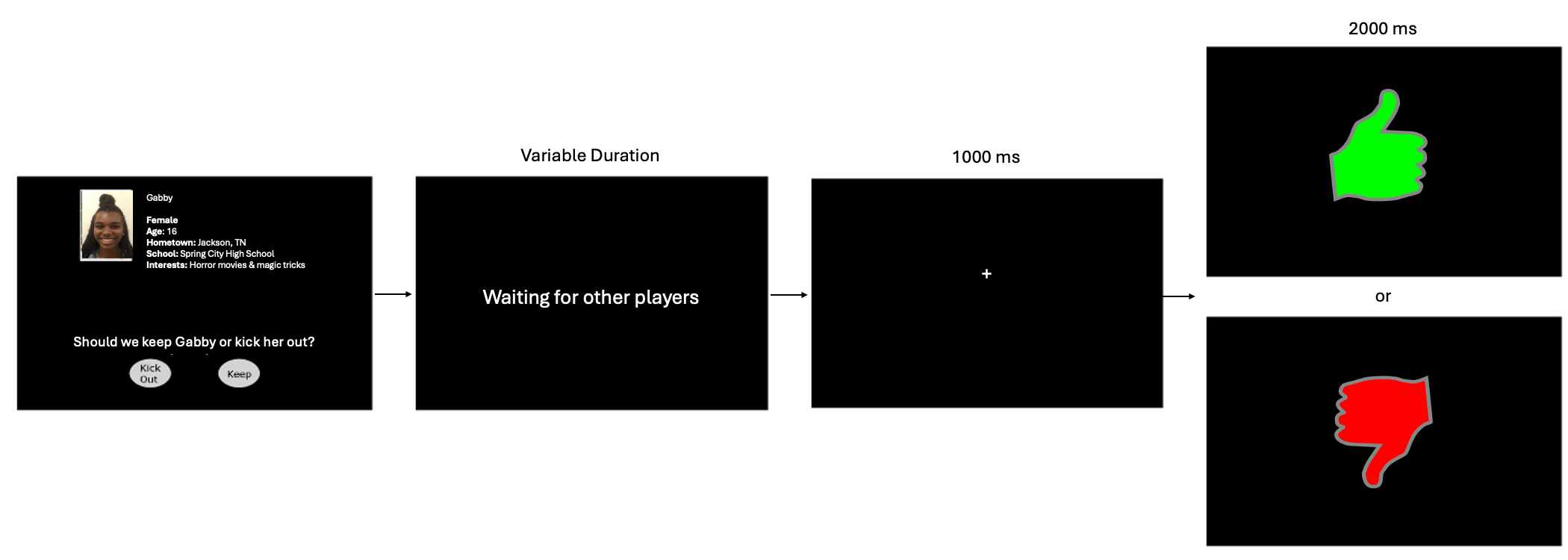


**Supplementary References**

Carskadon, M. A., & Acebo, C. (1993). A self-administered rating scale for pubertal development. *Journal of Adolescent Health*, *14*(3), 190–195. <https://doi.org/10.1016/1054-139X(93)90004-9>

Zbozinek, T. D., Rose, R. D., Wolitzky-Taylor, K. B., Sherbourne, C., Sullivan, G., Stein, M. B., Roy-Byrne, P. P., & Craske, M. G. (2012). Diagnostic Overlap of Generalized Anxiety Disorder and Major Depressive Disorder in a Primary Care Sample. *Depression and Anxiety*, *29*(12), 1065–1071. <https://doi.org/10.1002/da.22026>
